# Epigenomic profiling of immune cell subtypes reveals H3K27ac-marked stress signatures after long-duration spaceflight

**DOI:** 10.1038/s41598-025-17930-1

**Published:** 2025-09-12

**Authors:** Tabea L. Fullstone, Lukas F.J. Fischer, Maria Bohmeier, Petra Frings-Meuthen, Brian E. Crucian, Philipp Rathert

**Affiliations:** 1 Department of Molecular Biochemistry, Institute of Biochemistry, University of Stuttgart, 70569 Stuttgart, Germany; 2 German Aerospace Centre (DLR), Institute of Aerospace Medicine, Linder Hoehe, 51147 Cologne, Germany; 3 Johnson Space Center, National Aeronautics and Space Administration, Houston, TX USA

**Keywords:** Biochemistry, Molecular biology

## Abstract

Long-duration spaceflight imposes significant physiological stress on astronauts, including profound alterations in immune function. This study investigated epigenetic changes in immune cells following prolonged orbital spaceflight by analysing histone modifications in CD4+ and CD8+ T-cells from astronauts before, immediately after, and during recovery from spaceflight. Using Cleavage Under Targets and Tagmentation (Cut&Tag) to assess H3K27ac modifications, we identified significant alterations in chromatin accessibility, predominantly involving immune response pathways, gene regulation, and cellular adaptation mechanisms. While some epigenetic changes were transient, others persisted beyond 50 days post-return, suggesting long-term effects. These findings enhance our understanding of immune adaptation to spaceflight and have implications for mitigating spaceflight-associated health risks. Furthermore, they provide valuable insights into immune system regulation under high-stress conditions, potentially informing research on immunodeficiency disorders, cancer epigenetics, and aging-related immune decline on Earth. This study underscores the critical role of epigenetics in long-term space missions and terrestrial health applications.

**Supplementary Information:**

The online version contains supplementary material available at 10.1038/s41598-025-17930-1.

## Introduction

Humankind has been fascinated by the secrets of space for thousands of years. Long-term spaceflight exposes astronauts to unique exogenous and endogenous stressors including microgravity, radiation, isolation, and circadian disruption^1,2^. These processes impact astronauts in multiple ways affecting the brain, gut, and major organ systems including the immune system^2–4^. These chronic stressors exceed those experienced on Earth, leading to systemic changes that can have far-reaching and potentially irreversible effects. Among the most concerning consequences of prolonged exposure to space stress are the impacts on the immune system^5^, a complex network critical for maintaining homeostasis and defending the body against infections, inflammation, and diseases. Stress, whether physical, emotional or environmental, has been shown to activate the hypothalamic-pituitary-adrenal (HPA) axis and the sympathetic nervous system, leading to the release of glucocorticoids and catecholamines^6^. These stress hormones have a profound influence on immune function, altering immune cell signalling, proliferation and survival^7^. Acute stress may provide temporary immunological benefits by mobilizing immune cells to respond to immediate threats. However, under chronic stress conditions, as seen during long-term space missions, the immune system is severely dysregulated^5,8^. Prolonged activation of stress pathways leads to suppressed immune responses, chronic low-grade inflammation, and impaired immunosurveillance, all of which contribute to increased susceptibility to infections, latent virus reactivation and even autoimmune conditions^9^.

Importantly, chronic stress also induces epigenetic modifications that can have lasting effects on cellular functionality^10,11^. Epigenetic modifications, such as DNA methylation and histone acetylation, alter chromatin structure and help immune cells adapt to stress^12^. However, persistent stress can disrupt these regulatory mechanisms, leading to stable changes in gene expression that impair immune cell function^13^. For example, stress-induced changes in chromatin accessibility may suppress genes involved in immune activation and amplify pathways associated with inflammation, apoptosis, or cellular exhaustion^14^.

Spaceflight represents an ideal model for studying stress-induced epigenetic changes, as astronauts are exposed to a constellation of stressors over prolonged durations. Microgravity, in particular, reduces mechanical loading on cells, altering cellular pathways and inducing oxidative stress^15,16^, while radiation exposure triggers DNA damage and immune cell dysfunction. Psychological stress due to isolation, confinement, and disrupted circadian rhythms further exacerbates these effects^17,18^. Together, these factors create a chronic stress environment that uniquely challenges the immune system with consequences that may persist long after astronauts return to Earth. Previous studies have demonstrated that astronauts experience reduced T-cell function, dysregulated cytokine production and reactivation of latent viruses, such as Epstein-Barr virus and varicella-zoster virus, following spaceflight^5,9,19^. These findings highlight the vulnerability of the immune system to prolonged stress and suggest that such dysregulation may serve as a precursor to long-term health complications during space missions and on Earth.

The complex signalling pathways of the immune response are tightly regulated by epigenetic factors. Epigenetic changes play fundamental roles in biological and pathological processes by interpreting environmental signals and regulating gene expression. Apart from allowing the cells to adapt the immune response to different environmental inputs^20,21^, a dysregulation of the epigenome is associated with different pathologies including tumorigenesis. Immune cell function is often compromised in cancer and an effective immune response is not only influenced by factors like age or lifestyle but also by high stress levels^22–25^. The role of epigenetic modifications in mediating these changes remains underexplored but is of critical importance. Epigenetic alterations may serve as both markers and drivers of immune dysfunction, offering insights into how chronic stress leads to long-term immunological consequences.

In this study, we investigate the chromatin modification signatures of specific immune cell subtypes in astronauts following long-term spaceflight. In this context, we chose to focus on the histone modification H3K27ac (acetylation of lysine 27 on histone H3) because it is one of the most dynamic and responsive epigenetic marks. H3K27ac is associated with active promoters and enhancers and is known to rapidly reflect changes in gene expression following environmental stimuli, including stress. Due to its fast turnover, it serves as an early and sensitive marker for transcriptional activity changes, making it ideal for capturing the immediate epigenomic effects of long-term spaceflight on immune cells. Using a minimal cohort of three astronauts, we compared H3K27ac to those of a matched control cohort with no spaceflight exposure. Our analysis focuses on identifying stress-associated histone modification changes that influence gene expression patterns related to immune regulation, inflammation, and cellular function. We leveraged advanced epigenomic tools, such as Cut&Tag, to capture the molecular fingerprints of chronic stress on immune cells.

The findings of this study provide valuable insights into the long-term effects of stress on the immune system. Chronic immune dysregulation, if unresolved, has been linked to increased risks of autoimmune diseases, cardiovascular conditions, neurodegenerative disorders, and cancer. By identifying stress-induced chromatin modifications, we uncover key regulatory elements and biomarkers that may inform the development of targeted interventions to mitigate immune dysfunction.

## Results

**Fig. 1:**
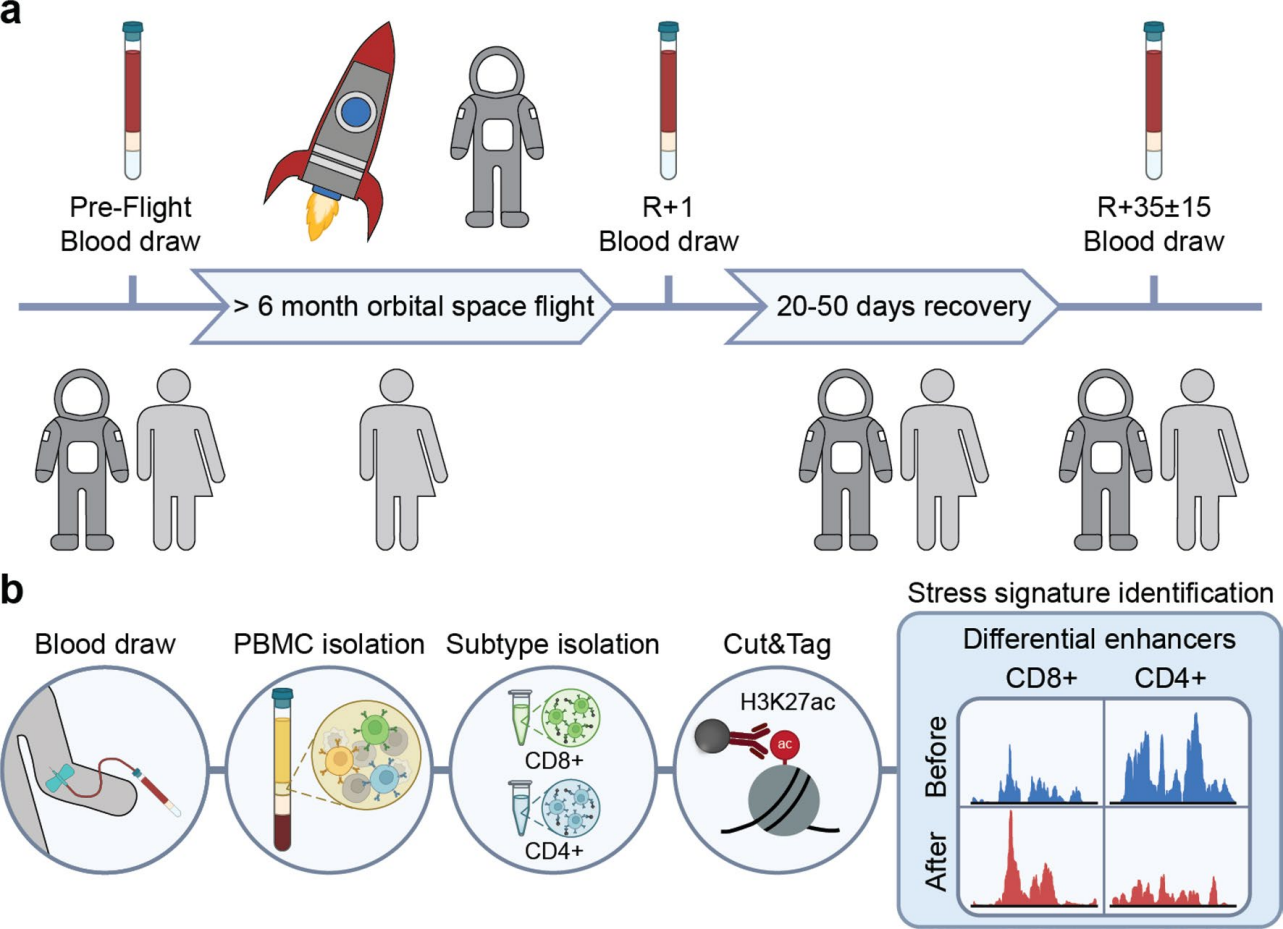
Schematic representation of the timeline and workflow of SpaceC&T to identify stress signatures in primary PBMCs. (**a**) Timeline of sample collection. (**b**) Workflow of sample processing, data collection and analysis.

To analyse epigenetic changes in immune cells upon high stress induced by prolonged orbital spaceflight, a cohort of three astronauts and control subjects was recruited consisting of 3 male astronauts between the age of 47–60 years and 3 male control subjects between the age of 30–38 years. The subjects donated blood at the start of the study (pre-flight sample, PF), after which astronauts spent > 6 months on the International Space Station (ISS). Upon return, astronauts underwent a blood draw within 24 h of return to achieve an on-return (R+1) sample. Control subjects underwent the R+ 1 blood draw ~ 8 months after the first blood draw. After a recovery period of 20–50 days post-return, a last blood draw was performed to yield the recovery (R+35 ±15) sample (Fig. 1a). To investigate possible changes in PBMC composition induced by prolonged orbital spaceflight, PBMCs were analysed for their subtype composition upon thawing using antibody staining. Subtype-specific epigenetic changes were determined in CD8+ (mainly cytotoxic T-cells) and CD4+ (mainly helper T-cells) immune cells by Cleavage Under Targets and Tagmentation (Cut&Tag) with an antibody directed against histone H3 Lysine 27 acetylation (H3K27ac). Following sequencing, regions with differential H3K27ac were determined from CD8+ and CD4+ immune cells to identify epigenetic stress signatures following prolonged orbital spaceflight (Fig. 1b).

### Prolonged orbital spaceflight does not influence PBMC subtype distribution

To identify possible changes in PBMC subtype composition following high stress induced by prolonged orbital spaceflight, an antibody panel was employed which is directed against CD45, CD3, CD56, CD4 and CD8 surface receptor proteins. This allows the identification of specific PBMC subtypes. Total PBMCs isolated from the whole blood of astronauts and control subjects were stained with this panel and analysed for their subtype composition by flow cytometry (Supplementary Fig. 1). For comparing the observed PBMC subtype composition values to expected values in healthy adults, reference ranges were sourced from publications with different study origins and the composition of PBMCs in all PF samples was compared to the expected literature values (Supplementary table 1). The distribution of PBMC subtypes within all PF samples was within the range of expected values, thus showing that the PBMC isolation and staining of PBMCs work reliably (Supplementary Fig. 2a). Interestingly, while some astronauts displayed slight alterations in PBMC composition, overall we did not observe any consistent and significant effects for the analysed cell types in astronauts and most changes were in the range of changes observed for the controls (Supplementary Fig. 2b-f).

### CUT&Tag for H3K27ac identifies major subtypes in PBMCs

**Fig. 2:**
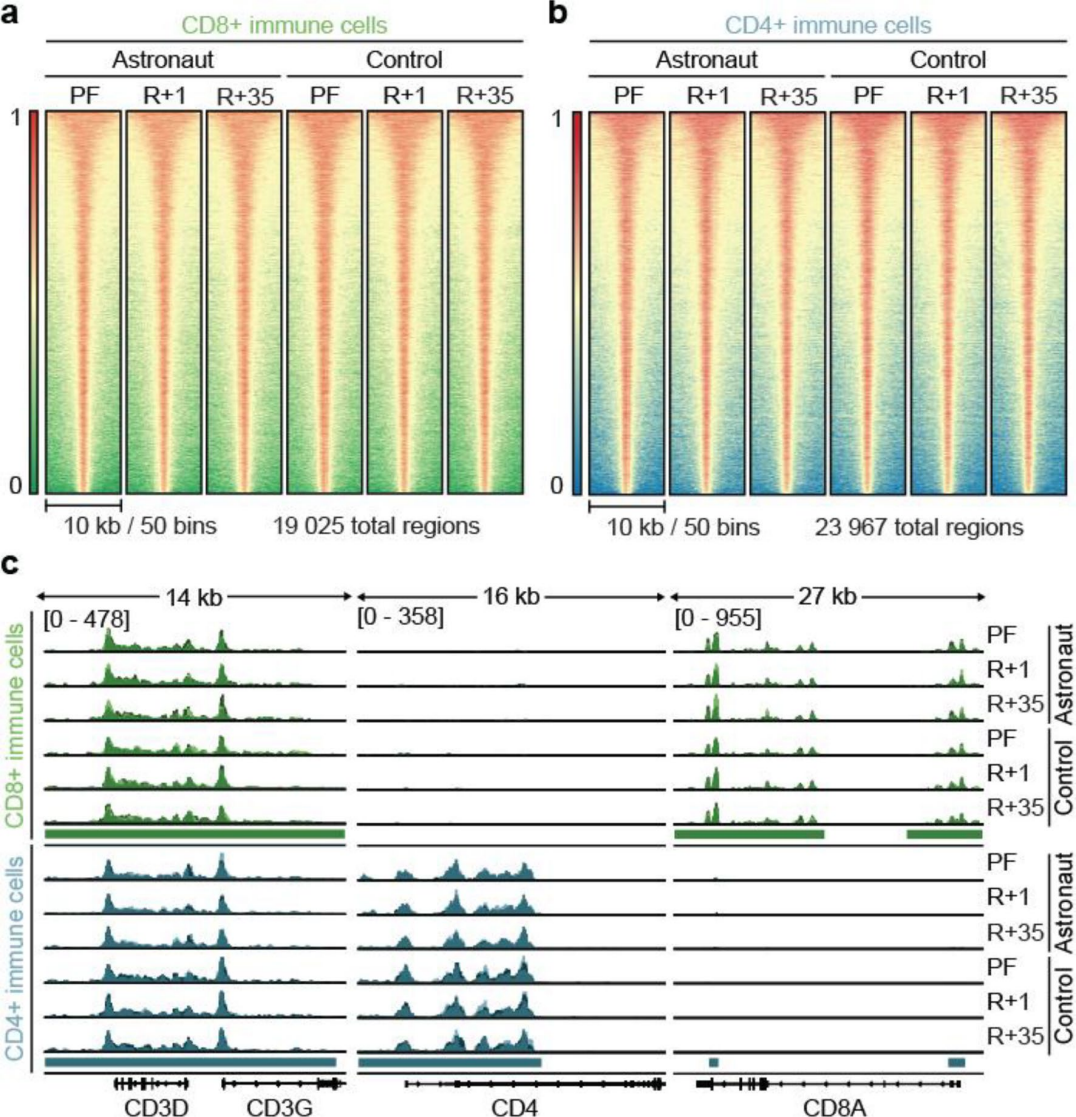
Characterisation of H3K27ac signal using Cut&Tag in primary PBMC subtypes following prolonged orbital spaceflight. (**a** and **b**) Heatmaps showing the average H3K27ac Cut&Tag signal for CD8+ (**a**) or CD4+ (**b**) immune cells. PBMC subtypes were extracted from total PBMCs of control subjects and astronauts pre-flight (PF) and post-flight at R+ 1 and R+35 ±15. CD8+ and CD4+ immune cells were then subjected to Cut&Tag for H3K27ac. For the generation of heatmaps, the replicates for each condition were combined into one bigwig file by averaging their signal. Signals are sorted by the average peak signal in descending order and plotted with a 10 kb window in 50 bins around the peak centre. (**c**) Representative Cut&Tag tracks of RPKM normalised H3K27ac intensity in CD8+ (green) and CD4+ (blue) immune cells at CD3D and CD3G, CD4 and CD8A from data in a and b. All samples were grouped to the same maximum signal indicated in the first line. Each replicate is shown in a different shade of blue or green and overlaid in the same track. *n* = 3.

After isolating selected PBMC subtypes and establishing conditions leading to improved Cut&Tag results, we investigated the epigenetic H3K27ac landscape in CD4+ and CD8+ immune cells. Due to the low cell numbers achieved, H3K27ac was analysed to identify promoters of actively transcribed genes and active enhancers using Cut&Tag, which opposed to ChIP-seq still yields high-quality data even for low cell numbers^26^. Cut&Tag led to the successful identification of 19 025 regions with H3K27ac signal in CD8+ immune cells (Fig. 2a) and 23 967 regions in CD4+ immune cells (Fig. 2b). Antibody staining confirmed that, while both cytotoxic T-cells and helper T-cells express CD3 on their surface, CD8 is a marker unique to CD8+ T-cells, whereas CD4 is only expressed on CD4+ T-cells (Supplementary Fig. 3). Indeed, both the CD3D and CD3G loci showed strong H3K27ac at the gene promoters in both cell isolations, whereas CD4 and CD8A only showed robust H3K27ac in their respective cell types (Fig. 2c).

### Prolonged orbital spaceflight leads to changes in H3K27ac

**Fig. 3:**
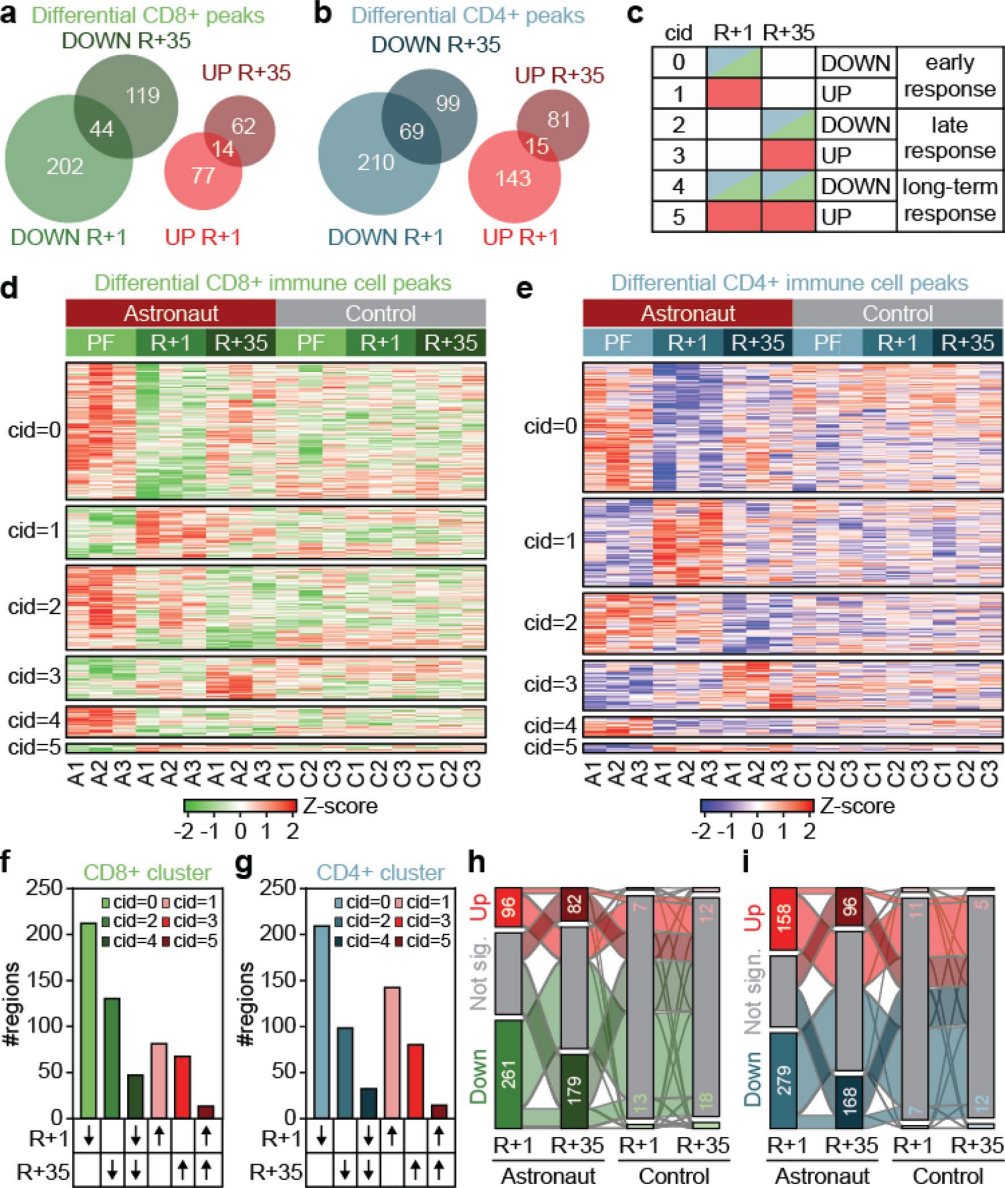
Identification of peaks with differential H3K27ac signal in CD8+ and CD4+ immune cells following prolonged orbital spaceflight. (a and b) Venn diagrams showing the overlap of differential regions with gain (red) or loss (blue/green) of H3K27ac signal in astronauts post-flight at R+1 and after a recovery period at R+35 ±15. Peaks with differential H3K27ac signal in CD8+ (**a**) and CD4+ (**b**) immune cells were identified using edge R. (**c**) Differential H3K27ac peaks from a and b were clustered into 6 clusters per cell type (cid = 0–5). Clustering was based on the effect following prolonged orbital spaceflight into early response (up/down in R+1), late response (up/down in R + 35) and prolonged response (up/down in R+1 and R + 35). (d and e) H3K27ac signal in differential clusters from A-C in CD8+ (**d**) and CD4+ (**e**) immune cells. Shown is the Z-score of the H3K27ac signal for astronauts (A1–A3) and control subjects (C1–C3) pre-flight (PF) and post-flight at R+1 and after a recovery period at R+35 ±15. (**f** and **g**) Number of differential H3K27ac peaks from d and e identified in CD8+ (**f**) and CD4+ (**g**) immune cells. (**h** and **i**) Alluvial plot showing transitions between differential H3K27ac peaks in clusters from F and G in CD8+ (**h**) and CD4+ (**i**) immune cells. The transition of peaks with gain in H3K27ac signal is marked in red and of peaks with loss of H3K27ac in green/blue. *n* = 3.

In both CD8+ and CD4+ cells, the number of regions with a loss of H3K27ac was higher compared to regions with a gain in H3K27ac (Fig. 3a,b). Not surprisingly, the number of differential regions was highest immediately upon return from spaceflight, but a considerable number of regions exhibited alterations during the recovery period (Fig. 3a,b). We clustered the differential peaks into regions that demonstrated early response (up/down in R+1), late response (up/down in R + 35) and long-term response (up/down in R+1 and R + 35) in order to distinguish between changes that occur during spaceflight but recover afterwards, changes that only occur upon return to Earth and changes that persist and could be permanent respectively (Fig. 3c-e). We observed the most profound changes in early response in both cell types analysed, followed by the number of changes with late response, while there were only very few regions with a long-term response (Fig. 3f,g). The identified regions were specifically changed due to microgravity conditions and prolonged stress during long-term orbital spaceflight, as the majority of regions with significant changes in H3K27ac signal in astronauts showed no changes in control subjects in both cell types (Fig. 3h,i).

**Fig. 4:**
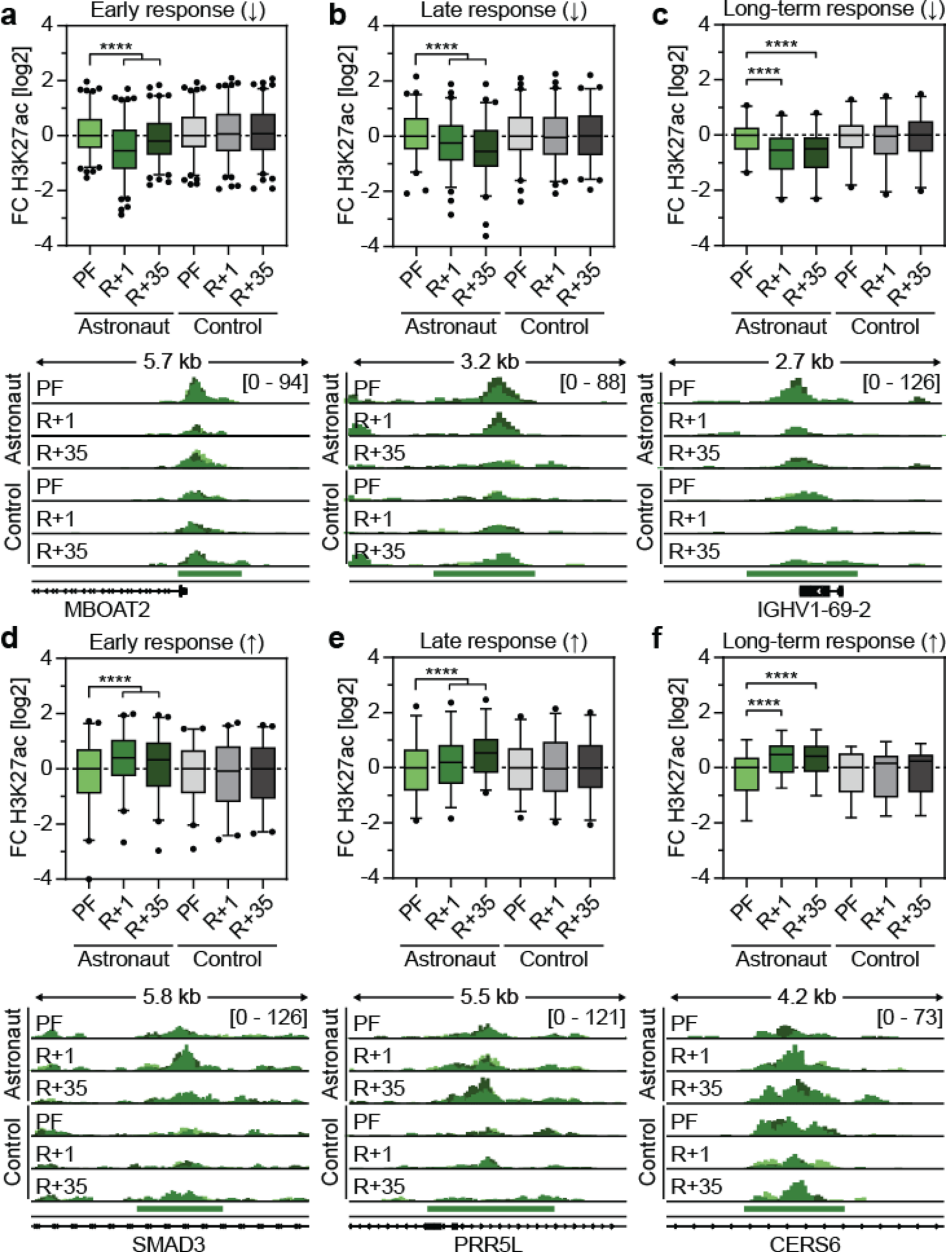
Astronauts experience early, late and long-term changes in H3K27ac in CD8+ immune cells upon prolonged orbital spaceflight. (**a**–**f**) Top: Boxplot showing changes in H3K27ac in CD8+ immune cells following prolonged orbital spaceflight. Shown are clusters with loss (a-c) or gain (d-f) in H3K27ac that show an early response/change in R+1 (**a**, **d**), late response/change in R + 35 (**b**, **e**) or long-term response/change in R+1 and R + 35 (**c**, **f**). H3K27ac signal of astronauts and control subjects pre-flight (PF) and post-flight at R+1 and R+35 ±15 was retrieved from bigwig files. The RPKM signal of the differential regions was averaged over each replicate subject and is displayed relative to the median of the PF sample in each group (astronaut or control). Box plot with 2.5 and 97.5 percentiles with individual data points shown only for values falling outside this interval, statistical analysis: Friedman test and Dunn’s multiple comparisons test (****: *p* ≤ 0.0001). Combined brackets signify same significance for all three comparisons (PF vs R+1 vs R + 35). Bottom: Representative Cut&Tag tracks of the boxplot of RPKM normalised H3K27ac intensity in CD8+ immune cells of control subjects and astronauts pre-flight and post-flight at R+1 and R+35 ±15. All samples were grouped to the same maximum signal indicated in the first line. Each replicate is shown in a different shade of green and overlaid on the same track. *n* = 3.

To investigate dynamics in H3K27ac in the differential clusters, the average H3K27ac signal was plotted for all identified differential regions in CD8+ immune cells (Fig. 4) and CD4+ immune cells (Supplementary Fig. 4). In CD8+ cells, regions with an early response showed the strongest effects on H3K27ac immediately after flight (R+1) as expected. However, after a period of 20–50 days (R + 35) the H3K27ac signal recovered only partly and was still significantly altered compared to pre-flight samples (Fig. 4a,d). While regions with a late response showed the strongest changes 20–50 days post-return (R + 35), they already exhibited significant yet milder changes upon return (R+1) (Fig. 4b,e). Finally, regions with long-term response were significantly changed upon return (R+1) with no recovery 20–50 days post-return (R + 35), suggesting that these changes could last much longer or may even represent permanent changes (Fig. 4c,f). While the strongest effects were always observed in the expected group (in R+1 for early response, in R + 35 for late response and in R+1 and R + 35 for long-term response), milder changes were also found for the other groups in astronauts. On the other hand, the control subjects showed no significant changes in the differential regions associated with prolonged orbital spaceflight (Fig. 4a-f).

The analysis of dynamic changes in H3K27ac in CD4+ immune cells (Supplementary Fig. 4) showed similar results compared to CD8+ immune cells (Fig. 4). While the strongest effects in the early response clusters were observed upon return (R+1) as expected, the clusters did not return completely to base level and still showed significant changes after 20–50 days of recovery (Supplementary Fig. 4a,d). The cluster containing late responding regions already showed mild but significant changes immediately after spaceflight (R+1), with the H3K27ac signal increasing even more 20–50 days post-return (R + 35) (Supplementary Fig. 4b,e). While control subjects only showed significant but biologically negligible changes in regions that lose H3K27ac immediately after flight (Supplementary Fig. 4a), they showed no significant changes for any of the other clusters (Supplementary Fig. 4b-f).

### Long-duration spaceflight induces deregulation of H3K27ac at gene promoters and putative enhancer elements

To analyse the differential regions associated with prolonged orbital spaceflight in more detail, the distance of the differential peaks relative to the transcription start site (TSS) of the nearest gene was analysed and compared to the frequency of all peaks as a baseline. Interestingly, regions with loss in H3K27ac were found close to the TSS (< 0.1–1 kb from TSS) much more frequently compared to all regions in both CD8+ immune cells and CD4+ immune cells (Supplementary Fig. 5a,b). This effect was independent of whether the H3K27ac loss occurred during spaceflight (↓ early response/R+1), after landing (↓ late response/R + 35) or was a long-term response to prolonged orbital spaceflight (↓ long-term response/R+1 and R + 35). In contrast, regions with gain in H3K27ac were found more frequently at distances of 5–10 kb or 10–50 kb away from the next TSS in both CD8+ and CD4+ cells (Supplementary Fig. 5a,b). As expected from their proximity to the TSS, regions with loss of H3K27ac induced during prolonged orbital spaceflight were mainly found at promoters and with a higher frequency than the baseline of all regions. Regions with gain in H3K27ac occurred with a higher frequency in introns and at intergenic regions of both CD8+ and CD4+ immune cells (Supplementary Fig. 5c,d). This is suggestive that prolonged orbital spaceflight leads to loss of H3K27ac mainly at promoters and a gain of H3K27ac at enhancer elements.

### Prolonged orbital spaceflight leads to an activation of the immune response in cytotoxic and helper T-cells

**Fig. 5:**
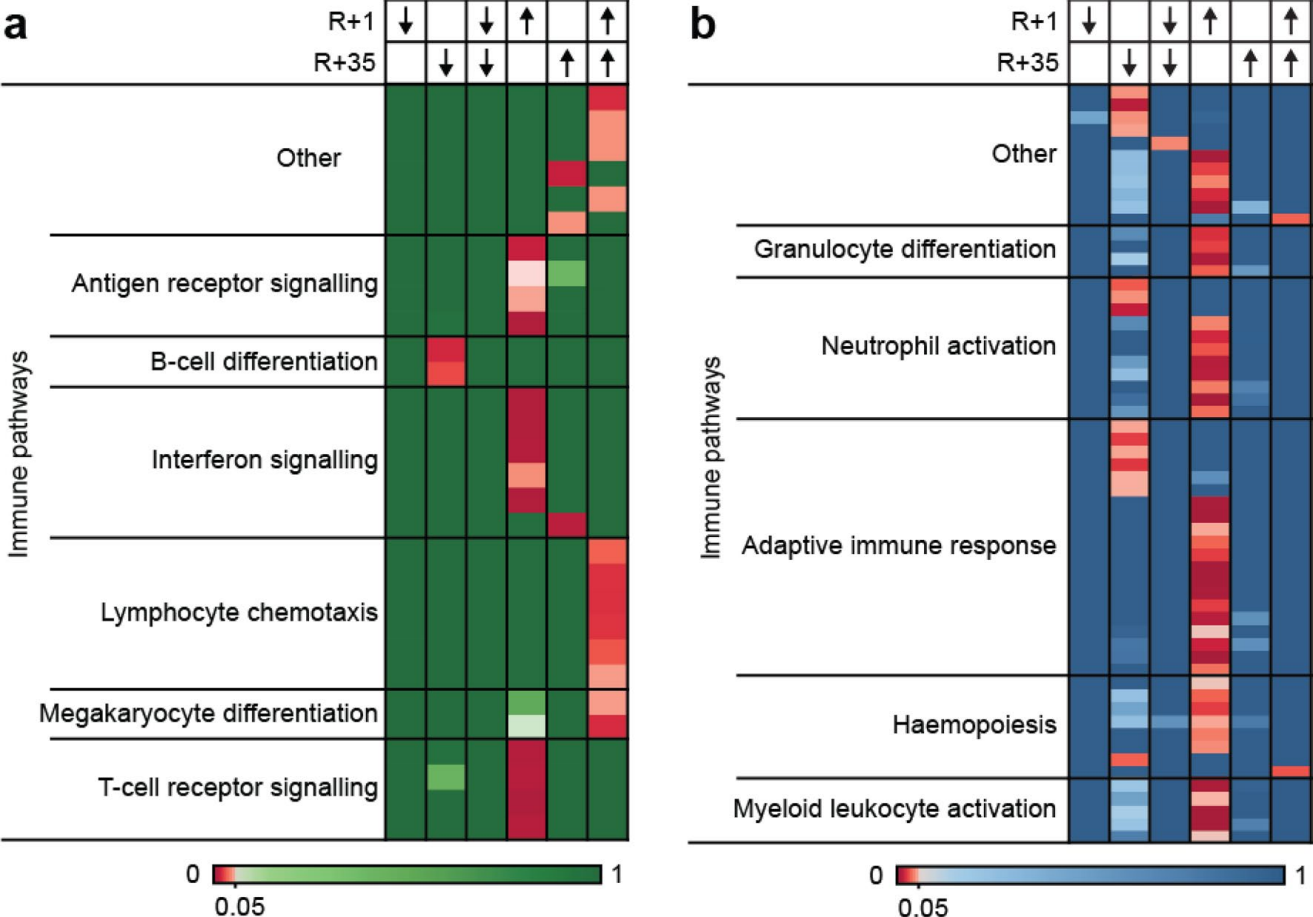
Prolonged orbital spaceflight induced a gain in H3K27ac in CD8+ and CD4+ immune cells at regions associated with immune pathways. (**a** and **b**) Gene-set-enrichment analysis for peaks with differential H3K27ac signal in CD8+ (**a**) and CD4+ (**b**) immune cells following prolonged orbital spaceflight. Analysis was performed for GO biological process pathways using ChIP-Enrich. All significantly enriched pathways (FDR ≤ 0.05) were categorised into immune-related pathways and further subcategorised using REVIGO.

**Fig. 6:**
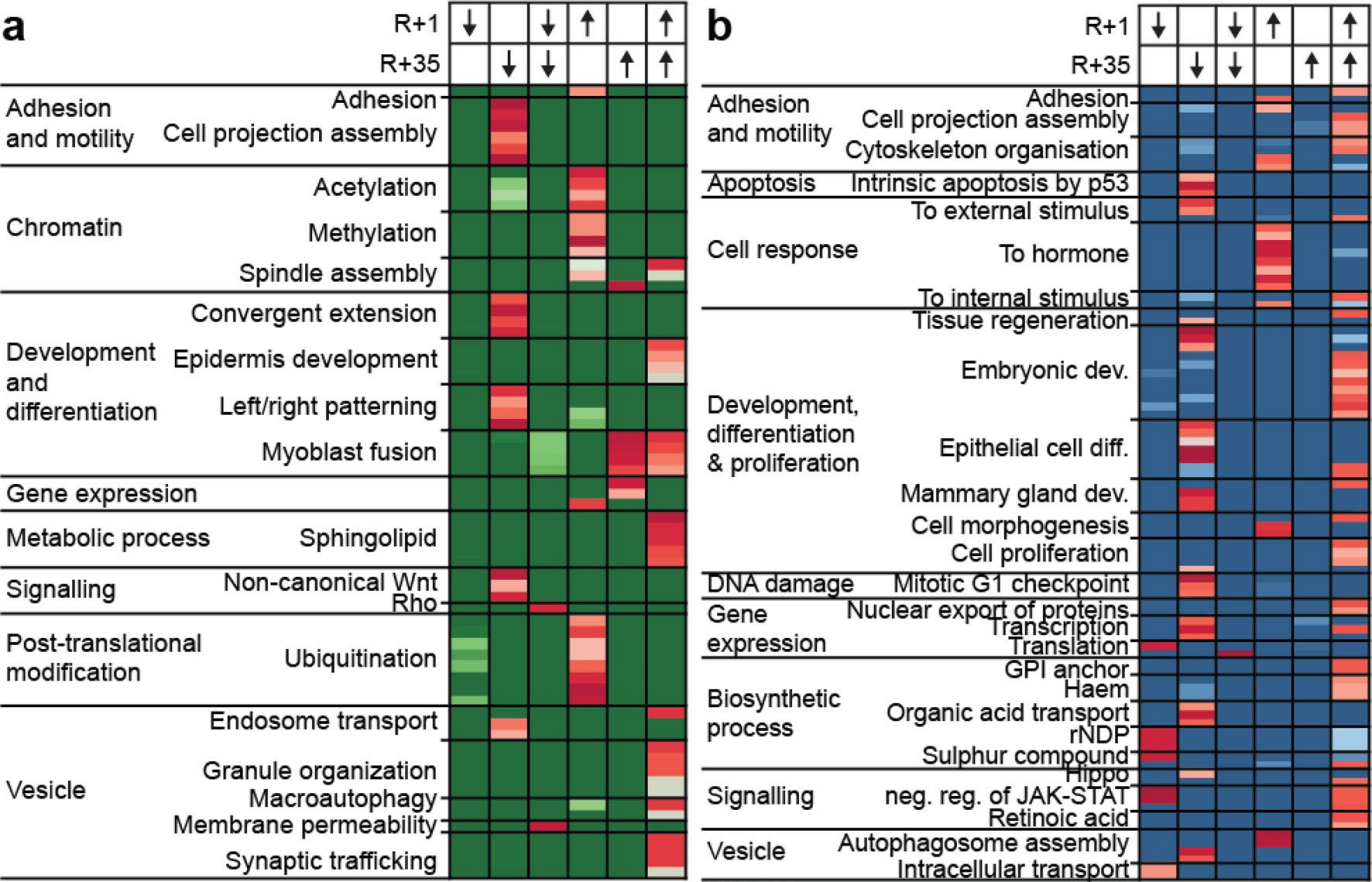
Prolonged orbital spaceflight induces changes in H3K27ac in in CD8+ and CD4+ immune cells at regions linked to diverse cellular signalling pathways. (**a** and **b**) Gene-set-enrichment analysis for peaks with differential H3K27ac signal in CD8+ (**a**) CD4+ (**b**) immune cells following prolonged orbital spaceflight. Analysis was performed for GO biological process pathways using ChIP-Enrich. All significantly enriched pathways (FDR ≤ 0.05) were categorised into non-immune-associated pathways and further subcategorised using REVIGO. Abbreviations: dev: development, diff: differentiation, neg: negative, reg: regulation.

To investigate pathways associated with the differential regions in both immune cell types, gene-set enrichment analysis was performed and all deregulated pathways (Supplementary table S1, S2) with significant enrichment were categorised depending on their function and combined into heatmaps (Figs. 5 and 6). Surprisingly, regions that lose H3K27ac in CD8+ immune cells during prolonged orbital spaceflight (↓ early response/R+1) were not significantly enriched for any pathways (Figs. 5a, 6a, Supplementary Fig. 6). Regions that lose H3K27ac during the 20–50 days post-return from space (↓ late response/R + 35), on the other hand, showed significant enrichment for immune pathways of B-cell differentiation (Fig. 5a, Supplementary Fig. 6). In addition, these late responding regions were also linked to pathways of cell projection assembly, non-canonical Wnt signalling, endosome transport as well as developmental pathways such as convergent extension and left/right pattern formation (Fig. 6a, Supplementary Fig. 6). Regions that lose H3K27ac during prolonged orbital spaceflight and that do not recover during the 20–50 days post-return (↓ long-term response/R+1 and R + 35) were only enriched for negative regulation of membrane permeability and Rho signalling (Fig. 6a, Supplementary Fig. 6).

The number of significantly enriched pathways of regions with a gain of H3K27ac during prolonged spaceflight was higher in comparison to regions that lose H3K27ac. Prolonged orbital spaceflight (↑ early response/R+1) increased H3K27ac at regions associated with several immune response pathways, including signalling via antigen receptors, cell surface receptors, T-cell receptors and interferon (Fig. 5a, Supplementary Fig. 6). Furthermore, prolonged orbital spaceflight also induced a gain of H3K27ac at regions that are linked with post-translational modification pathways, including histone acetylation and methylation, as well as protein ubiquitination (Fig. 6a, Supplementary Fig. 6). Regions that only gain H3K27ac during the 20–50 days post-return (↑ late response/R + 35) were mainly implicated in gene expression pathways, as well as in myoblast fusion and syncytium formation (Fig. 6a, Supplementary Fig. 6). Finally, regions that gain H3K27ac and do not recover during the recovery period (↑ long-term response/R+1 and R + 35) were enriched for immune pathways of lymphocyte chemotaxis and megakaryocyte differentiation (Fig. 5a, Supplementary Fig. 6) and pathways linked to myoblast fusion, vesicle organisation and trafficking, epidermis development and sphingolipid metabolic process (Fig. 6a).

Similar to CD8+ immune cells, a large range of the regions that gain H3K27ac in CD4+ immune cells during spaceflight (↑ early response/R+1) are connected to immune pathways, such as granulocyte differentiation, haemopoiesis, neutrophil and myeloid leukocyte activation, as well as pathways belonging to the adaptive immune response, including signalling via Fc receptors, antigen receptors, Toll-like receptor or cell surface receptors (Fig. 5b, Supplementary Fig. 7). Other than in CD8+ immune cells, in CD4+ cells these immune pathways were not solely connected to regions that gain H3K27ac during spaceflight, but also to regions that lose H3K27ac during the 20–50 days post-return (↓ late response/R + 35), which showed enrichment for neutrophil activation and adaptive immune response/T-cell mediated immunity pathways (Fig. 5b, Supplementary Fig. 7).

When focusing on non-immune pathways, the differential regions of CD4+ immune cells again did not cluster as well into categories as the regions of CD8+ immune cells (Fig. 6a) but showed a more diverse pattern (Fig. 6b). Regions that lose H3K27ac during prolonged orbital spaceflight (↓ early response/R+1) were mainly enriched for negative regulation of receptor signalling via JAK/STAT, nucleoside bisphosphate metabolism and intracellular vesicle transport (Fig. 6b, Supplementary Fig. 7). Regions that only lose H3K27ac after the astronauts have returned to Earth (↓ late response/R+1) were mainly associated with apoptosis, DNA damage, response to external stimuli, transcription and the transport of organic acids, but were also in part connected to embryonic development, as well as epithelial cell differentiation pathways (Fig. 6b, Supplementary Fig. 7). Similar to CD8+ cells (Figs. 5 and 6), regions with long-term loss of H3K27ac in CD4+ immune cells were only significantly enriched in two pathways, namely the regulation of translational fidelity and the cellular defence response pathway (Fig. 6b). Apart from their connection to immune pathways, regions that gain H3K27ac during prolonged orbital spaceflight (↑ early response/R+1) were mainly enriched for pathways of cytoskeleton organisation, response to hormone and cell morphogenesis (Fig. 6b). While regions with long-term gain of H3K27ac were only connected to two immune pathways, they were linked to many other pathways, among them nuclear protein export, biosynthetic processes, as well as retinoic acid signalling and negative regulation JAK/STAT signalling. Furthermore, they were also enriched for pathways of cell projection assembly, cytoskeleton organisation, embryonic development and cell proliferation (Fig. 6b).

Our findings demonstrate that while prolonged orbital spaceflight does not significantly alter PBMC subtype distribution, it induces extensive alterations in H3K27ac-marked genomic regions in immune cells, particularly regions associated with immune regulation, gene expression, and cellular adaptation. The observed changes suggest an early and transient response to spaceflight stress, with some alterations persisting beyond the recovery period, potentially indicating long-term epigenetic imprinting.

## Discussion

To date, many studies and reviews have been published that analyse the effect of space missions on the immune system of astronauts^5,9,27,28^. While most studies focus on changes in the composition and activation capability of immune cells following spaceflight^29–31^, only recently studies have started to emerge that aim to analyse the transcriptome of immune cells following spaceflight or simulated microgravity to identify changes in gene expression underlying the observed immune system alterations^32,33^. Only in 2024, the repertoire has been further extended by the addition of single-cell RNA-seq (Kim 2024; Wu 2024). The first study that also included the analysis of epigenome changes following spaceflight was the NASA twin study. This multidimensional study combined biomedical and multi-omics data to analyse changes in a twin following 12 months of spaceflight compared to the earthbound control twin (Garrett-Bakelman 2019). This study analysed epigenome changes in the form of DNA methylation changes for the first time, however, its major drawback is the sample size of one, making extensive validations necessary. A first attempt in this regard was undertaken by Kim and colleagues who analysed changes in transcription and chromatin accessibility on a single-cell level following short-duration spaceflight^34^. In an effort to close the gap in the characterisation of epigenome changes following long-duration space missions, we established a workflow for the extraction of total PBMCs, subtype isolation and analysis of H3K27ac changes following prolonged orbital spaceflight (Fig. 1). The established workflow allowed for the first time to analyse changes in H3K27ac in CD4+ and CD8+ immune cells of astronauts following long-duration spaceflight. The application of this workflow identified a rearrangement of H3K27ac at promoters and putative enhancer elements in CD4+ and CD8+ immune cells following spaceflight. The affected regions are associated with immune, developmental, metabolic and biosynthetic pathways, suggesting that long-duration spaceflight influences the expression of the associated genes via deregulation in H3K27ac. In line with previous data^35^, we observed only mild changes in PBMC subtype composition and the control group displayed similar changes (Supplementary Fig. 2), suggesting that the inherent experimental variation is higher than the changes caused by prolonged spaceflight. Furthermore, due to the limited sample size and the variability in the observed effects, we are unable to make definitive claims about the influence of prolonged spaceflight on PBMC distribution. Contrary to these findings, short-duration spaceflight (~ 2 weeks) was shown to induce significant alterations in PBMC subtype composition^29,36^. A potential explanation for this effect could be the countermeasure-based improvements of physiological wellbeing of astronauts that started to emerge around 2012^9^. Operational upgrades and biomedical countermeasures aboard such as increased resupply missions, improved personal communication, upgraded exercise equipment and routines, enhanced food quality and nutritional support had a beneficial impact on mitigating spaceflight-related immune dysregulation^37^. The follow-up analysis of H3K27ac changes in CD4+ and CD8+ immune cells by Cut&Tag^26,38–40^ revealed that long-duration spaceflight induced changes in H3K27ac at distinct genomic loci. While some of the changes induced by long-duration spaceflight bounced back during the recovery period, a small subset did not recover their H3K27ac signal and a lot of changes only fully manifested themselves after the recovery period (Figs. 2, 3 and 4, Supplementary Fig. 4) which is in agreement with previous studies in cell lines exposed to simulated microgravity^33,41^ and short-term missions^34^. Similar delayed effects have also been observed in mice, where reductions in B lymphopoiesis were reported one week after landing^42^. However, although mice share genetic similarities with humans, physiological and molecular differences can limit their translational relevance. In particular, murine transcriptional responses to inflammatory stimuli have been shown to differ markedly from those in humans^43^, underscoring the importance of validating such findings in human studies.

Enrichment analysis of the differential H3K27ac peaks in CD8+ immune cells revealed that long-duration spaceflight led to a reduction of H3K27ac at regions linked to genes of B-cell differentiation with a concomitant increase of H3K27ac at regions associated with antigen receptor, T-cell receptor and interferon signalling (Fig. 5). Interestingly, while the changes in H3K27ac associated with these pathways recovered during re-adaptation to the terrestrial environment, long-duration spaceflight also led to increased H3K27ac at regions linked to lymphocyte chemotaxis and megakaryocyte differentiation, which did not recover during the recovery period. Megakaryocytes are involved in haemopoiesis where they are responsible for the production of blood platelets^44^ and previous studies showed that irradiation increases the differentiation of megakaryocytes both in rats and human cell lines^45,46^. The observed increase in megakaryocyte differentiation could be caused by the space radiation astronauts experience during their missions.

In CD4+ immune cells long-duration spaceflight did not induce long-term changes but mainly induced a transient increase in H3K27ac at immune pathways (Fig. 6) such as haemopoiesis, neutrophil and myeloid leukocyte activation as well as granulocyte differentiation. Anaemia is a long-known effect of spaceflight on the human body and was shown to be caused by increased haemolysis in space^47^ and a subsequent increase in leukocytes and erythrocytes^35,48^, which is most likely a compensatory mechanism to counterbalance the haemolysis induced by spaceflight. The increase in neutrophil levels was previously only described in short-term missions^49–51^ and the presented data suggests this might also be the case for long-term missions. Overall, the changes in H3K27ac observed at immune pathway genes are mainly associated with an activation of the immune response (Fig. 6). Astronauts frequently experience reactivation of latent viruses (Cowen 2024; Mehta 2017; Rooney 2019) and dysregulation in cytokine levels (Crucian 2014; Gertz 2020; Kim 2024; Krieger 2021; Morukov 2011), which could be the reason for the upregulation of these pathways in our data.

Besides immune pathways, CD4+ and CD8+ immune cells also showed changes in H3K27ac in regions associated with cell adhesion, cell motility, gene expression, signalling, vesicle trafficking as well as metabolic and biosynthetic process pathways, which suggests that this is a response to the exposure to microgravity for a prolonged period of time. Interestingly, CD8+ immune cells displayed an increase in H3K27ac at regions associated with chromatin modifications such as acetylation and methylation following return to Earth, which could explain why some of the changes observed only arise during acclimation to the terrestrial habitat (Fig. 5). This suggests that the persistence of epigenetic alterations may indicate long-term remodelling of chromatin architecture, potentially affecting gene expression programs critical for immune function, cellular repair, and adaptation processes. This could have implications for the long-term health of astronauts, including an increased susceptibility to immune dysregulation and related diseases.

CD4+ immune cells also showed an increase in H3K27ac at regions linked to cellular hormone response (Fig. 6), which was previously described^52,53^. Notably, spaceflight induced an upregulation of H3K27ac at regions linked to the negative regulation of JAK-STAT signalling and this upregulation did not subside during the recovery period. JAK-STAT signalling has been described as a “central communication node of the immune system”^54^ and plays important functions in the development, differentiation, function and homeostasis of T-cells^55–58^. Experiments in simulated microgravity suggest that there are changes in the distribution of the CD4 T-cell subtypes^59^ and a reduction in T-cell function^27,30,35^, which might at least in part be driven by the increased inhibition of JAK-STAT signalling following spaceflight.

Finally, spaceflight also induced changes in H3K27ac in CD4+ and CD8+ immune cells at pathways connected to development and differentiation (Figs. 5 and 6). Both cell types displayed changes in pathways associated with skin development and regeneration, such as epidermis development and epithelial cell differentiation. This is in line with studies showing that spaceflight is implicated in epidermis thinning, altered wound healing, skin rashes and irritation^60,61^. CD8+ immune cells also displayed an increase in H3K27ac at pathways linked to myoblast fusion (Fig. 5). Myoblast fusion is an important event in the development, regeneration and repair of muscle fibres^62^ and CD8+ T-cells were shown to play an important role in muscle regeneration and repair^63,64^. Even though astronauts undergo a rigorous exercise program during their time in microgravity, prolonged spaceflight still leads to a reduction in muscle fibre density^65^, suggesting that epigenetic changes help orchestrate muscle repair during long-duration spaceflight.

Lastly, long-duration spaceflight also led to the deregulation of pathways important during embryonic development such as convergent extension, left/right patterning, mammary gland development and others (Figs. 5 and 6). Interestingly, changes in these pathways either occurred during re-adaptation to Earth or immediately following return, but without recovery. This is most likely an effect induced by the long-term exposure to microgravity which disrupts the cytoskeletal equilibrium. This has been shown previously in human cell lines which adapt to microgravity within seconds of disorientation, triggering a cascade of cellular responses, including morphological alterations, changes in mechanical properties, extracellular matrix reorganization, and the activation or inhibition of various signalling pathways^66^. These interconnected processes ultimately result in functional adaptations of cells to the microgravity environment of space^67^. It was recently shown that tissue damage can lead to the reactivation of embryonic gene programs, which provide the cells with transcriptional plasticity to promote tissue regeneration. When this situation is not resolved and embryonic gene expression is induced long-term, this can lead to the manifestation of diseases including cancer^68^. Indeed, several studies showed that tumorigenesis is associated with the dysregulation of pathways important in embryonic development^69,70^ and spaceflight has already been implicated in the altered expression of cancer-related genes in lymphocytes^36^. Therefore, a prolonged dysregulation in embryonic signatures could be associated with an increased risk for astronauts to develop cancer and should be monitored closely.

The importance of epigenetic regulation of gene expression programs during long-duration spaceflight is becoming increasingly apparent and more and more studies are starting to emerge that analyse the gene regulatory changes underlying the observed changes in the bodies of astronauts following space missions. Recently, studies started to emerge that identified changes in the expression of long non-coding RNAs following spaceflight^71^, the effect of simulated microgravity on DNA methylation in human lymphoblastoid cells^72^ and changes in N6-methyladenosine profiles of whole blood of astronauts following spaceflight^73^. This highlights the need for further epigenome studies investigating the effect of long-duration space missions on the epigenome of astronauts.

As with any study involving unique and hard-to-access populations, certain limitations must be acknowledged. The small number of participants (n = 3) reflects the logistical and practical constraints of conducting research in the context of human spaceflight. Additionally, there is an age difference between the astronaut group and the controls, and astronauts inherently represent a distinct and highly selected subset of the population. To mitigate these factors, we focused our analysis on dynamic changes in H3K27ac signals within the astronaut group across timepoints, rather than comparing baseline levels between groups. Moreover, we filtered for regions that showed changes exclusively in astronauts and not in controls, aiming to isolate the epigenetic effects of spaceflight from unrelated variability. One additional limitation of our study is the timing of the first post-flight blood collection, which occurred approximately 24 h after landing. It is possible that some transient immune changes may have already begun to normalize during this period, potentially dampening the observable effects of spaceflight in our post-landing samples. We further acknowledge that the timing of the post-landing blood draw, which varied slightly due to operational constraints, may have introduced minor variability.

The established workflow provides the framework to further investigate the impact of epigenetic changes on the immune system of astronauts and could easily be expanded to include additional epigenetic modifications or chromatin interactions, allowing for a comprehensive multi-omics analysis of the changes occurring in immune cells following long-duration space missions. These results provide valuable insights into the molecular adaptations of the immune system under spaceflight conditions and highlight the need for further investigation into the long-term health implications for astronauts. Moreover, the parallels between spaceflight-induced immune changes and immune dysregulation in aging, cancer, and autoimmune diseases suggest broader terrestrial relevance. Thus, the impact of this study extends beyond space medicine, offering novel perspectives on the intricate relationship between epigenetics, immune regulation, and disease susceptibility. Future studies should aim to elucidate the functional consequences of these epigenetic changes and explore potential countermeasures to mitigate the impact of spaceflight on immune health, ensuring the success of long-duration missions and benefiting medical research on Earth.

Ultimately, this work sheds light on the profound and potentially irreversible effects of prolonged stress on immune function. Our findings have far-reaching implications not only for long-term deep space missions but also for terrestrial populations exposed to chronic stress, such as individuals facing aging, trauma or chronic illness. By elucidating the molecular connections between stress, epigenetics and immune dysfunction, this study represents a foundational step toward improving health outcomes in individuals subjected to prolonged stress environments.

## Methods

### Antibodies

CD56-PE-Vio770 (Miltenyi Biotec Bergisch Gladbach, Germany; 130–113-313, clone REA196), CD3-PE (Miltenyi Biotec Bergisch Gladbach, Germany; 130–113-139, clone REA613), CD4-VioBlue (Miltenyi Biotec Bergisch Gladbach, Germany; 130–114-534, clone REA623), CD8-FITC (Miltenyi Biotec Bergisch Gladbach, Germany; 130–110-677, clone REA734), CD45-APC (Miltenyi Biotec Bergisch Gladbach, Germany; 130–110-633, clone REA747) and H3K27ac Rabbit mAb (Cell Signaling Technology, #8173).

### Subject cohort and ethics approval

A total of 3 male astronauts and 3 control subjects were recruited for the project. Astronauts were between the age of 47 – 60 years and control subjects between the age of 30 – 38 years. For this study astronauts from long-duration space missions were picked and all astronauts spent ~ 6 months (here 157 or 170 days) on the International Space Station (ISS). The study has been approved by the ESA Medical Board, the Human Research Multilateral Review Board (HRMRB) of NASA and the Commission for Responsibility in Research (Ethics Commission) of the University of Stuttgart (reference number Az. 20–007). All subjects gave their written informed consent and research was performed in accordance with the Declaration of Helsinki. Samples were anonymised for data analysis.

### Blood sample collection and PBMC isolation

Blood samples for astronauts were collected at the following time points: 55 – 185 days before flight (pre-flight, PF), within the first 24 h after landing (R+1) and 20–50 days after landing (R+35 ±15). Blood draw for control subjects was performed at the following time points: at the beginning of the study (PF), after 251 days (R+1) and between 20–50 days after the R+1 blood draw (R+35 ±15). To minimize variability, all blood samples from astronauts were collected in the morning after a 5-h fast, helping to reduce circadian and metabolic influences. From each subject, 3 × 8 ml of whole blood was collected directly into BD Vacutainer® CPT™ Cell Preparation Tube with Sodium Citrate (BD Bioscience, Franklin Lakes, US; 362,761) to prevent coagulation. Mononuclear cells were isolated from whole blood according to manufacturer instructions. In brief, following blood draw Vacutainer® CPT™ tubes were gently inverted 8 – 10 times. Within 2 h following blood draw, samples were gently inverted 8 to 10 times again and then centrifuged at 1500 × g at room temperature for 30 min to achieve separation of red blood cells and granulocytes from PBMCs and platelets. The PBMC layer was transferred into a 50 ml centrifuge tube and resuspended in 50 ml PBS. All following centrifugation steps were carried out at room temperature. PBMCs were pelleted by centrifugation at 300 × g for 10 min. Cells were washed once with 50 ml PBS and once with 30 ml PBS, and both times cells were pelleted by centrifugation at 200 × g for 12 min. Finally, PBMCs were resuspended in 1 ml cryofluid (90% FBS, 10% DMSO) and stored at -80 °C until use.

### Separation of subtype from total PBMCs

For the isolation of PBMC subtypes for H3K27ac Cut&Tag, an HDAC inhibitor cocktail (HDACic) was prepared containing 50 µM Trichostatin A (Sigma-Aldrich Burlington, US; T1952) and 500 mM sodium butyrate (Sigma-Aldrich Burlington, US; 8.17500). In addition, cell separation buffer (CSB) was prepared containing 0.5% BSA, 2 mM EDTA and 0.09% sodium azide. The CSB buffer was supplemented 1:100 with HDACic (CSB+) to avoid loss of H3K27ac and this CSB+ was used for all steps of subtype isolation unless further specified. Frozen PBMCs were thawed in 10 ml PBS supplemented with HDACic (1:100) and following centrifugation at room temperature and 300 × g for 10 min, the cell pellet was resuspended in 1 ml CSB+ . CD56+ , CD8+ and CD4+ immune cells were isolated from total PBMCs in sequential order using magnetic microbeads for CD56, CD8 and CD4 respectively (Miltenyi Biotec, 130–050-401, 130–045-101 and 130–045-201) following manufacturer instructions. In brief, cells were incubated with microbeads for 15 min at 2 – 8 °C. Cells were then washed using CSB+ and loaded onto an equilibrated MACS MS column (Miltenyi Biotec, 130–042-201) in the magnetic field of the OctoMACS™ Separator (Miltenyi Biotec, Bergisch Gladbach Germany; 130–042-109). Cells were washed three times with CSB+ and the flow-through was collected for the next round of cell extractions. After three washes, labelled cells were eluted from the column and cell count was determined by flow cytometry using Muse® Count & Viability Kit (Cytek Biosciences Fremont, US; MCH100102).

### Cell surface staining of PBMCs

To analyse PBMC subtype distribution in total PBMCs and to verify the successful isolation of PBMC subtypes using magnetic microbeads, cells were stained with an antibody panel to distinguish live PBMCs, total T-cells, NK cells, NK T-cells, helper T-cells and cytotoxic T-cells. For antibody staining of cell surface receptors, 5 × 10^4^ total PBMCs or 2 × 10^4^ isolated PBMC subtypes were transferred to a 96-well U-bottom plate, centrifuged and supernatant taken off. All centrifugation steps were performed at 300 × g for 5 min at room temperature. Viobility dye (Miltenyi Biotec Bergisch Gladbach, Germany; 130–130-421) was prediluted 1:100 in PBS^Mg2+ Ca2+^, cells were resuspended in 100 µl diluted Viobility dye and incubated for 15 min at room temperature. Following incubation, samples were washed by the addition of 100 µl CSB, resuspension and centrifugation. During centrifugation, fluorochrome-coupled primary antibodies CD56-PE-Vio770, CD3-PE, CD4-VioBlue, CD8-FITC and CD45-APC were diluted 1:50 in CSB. After centrifugation, the supernatant was removed and cells were resuspended in 100 µl diluted antibodies. Following incubation for 20 min at 2 – 8 °C protected from light, cells were washed by the addition of 100 µl CSB as described above. Following washing, cells were fixed in 100 µl 4% PFA for 15 min at room temperature in the dark. After a last washing step using CSB, cells were resuspended in 150 µl CSB and analysed by flow cytometry.

### Cut&Tag

Cut&Tag on isolated CD8+ and CD4+ immune cells was performed using the CUT&Tag Assay Kit (Cell Signaling Technology Danvers, US; 77,552) according to manufacturer instructions with the following changes: (1) HDACic was added 1:100 to all buffers that come in contact with cells to avoid loss of H3K27ac and (2) Digitonin solution was replaced by the addition of 5% Triton-X-100 to a final concentration of 0.05% in the buffers to improve permeabilisation. In brief, 3—10 × 104 cells were washed, bound to activated Concanavalin A beads and then incubated with 1 µl H3K27ac antibody (Cell Signaling Technology, 8173) at 4 °C overnight. Following incubation with a secondary rabbit antibody, pAG-Tn5 transposase was recruited to chromatin-bound antibodies and Tn5 was activated by the addition of Mg2+ at 37 °C to start tagmentation and adaptor ligation. Subsequently, chromatin was released from the cells, the tagmented DNA purified and Cut&Tag libraries were constructed using the CUT&Tag Dual Index Primers and PCR Master Mix for Illumina Systems (Cell Signaling Technology Danvers, US; 47415S) according to manufacturer instructions. Sequencing of the Cut&Tag libraries was performed by Novogene on an Illumina NovaSeq 6000 deep sequencer acquiring 2 × 150 bp paired-end reads aiming at 2.5 mio paired-end reads (5 mio total reads) per sample.

### Analysis of Cut&Tag data

Cut&Tag data was analysed using the Galaxy web platform (https://usegalaxy.eu/). Quality of sequencing data was assessed using FastQC (Galaxy Version 0.74 + galaxy0) (Andrews 2010). Low-quality bases and adapter contaminations were removed using Trim Galore! (Galaxy Version 0.6.7 + galaxy0). Data was mapped to the human reference genome hs1 (T2T CHM13v2.0) using Bowtie2 (Galaxy Version 2.5.3 + galaxy1)^74^. Peak-calling was performed using the Sparse Enrichment Analysis for CUT&RUN (SEACR) tool (Galaxy Version 1.3 + galaxy1)^75^. SEACR was used in the “stringent” mode with the “norm” flag set and control type set to “Threshold” with a threshold of 0.03. BED files were further processed using the bedtools suite^76^ on Galaxy. All peaks called in CD4+ or CD8+ immune cells respectively were concatenated using Concatenate datasets (Galaxy Version 0.1.1). After sorting the BED files using bedtools SortBED (Galaxy Version 2.30.0 + galaxy2), nearby and overlapping intervals were merged using bedtools MergeBED (Galaxy Version 2.30.0). The two merged BED files containing all peaks of CD4+ or CD8+ immune cells respectively were used for downstream analysis.

For visualisation of Cut&Tag tracks, bigwig files were averaged over replicates using bigwigAverage (Galaxy Version 3.5.4 + galaxy0) from the deepTools2 suite^77^. Heatmaps were generated using the Chromatin Analysis and Exploration (ChAse) tool version 1.1.2^78^ from the averaged bigwig files and the merged bed files for each immune cell type with a 10 kb window in 50 bins around the peak centre. Visualisation of ChIP-seq tracks was done using the Integrative Genomics Viewer (IGV) version 2.13.2^79^. For identification of differential peaks, the average H3K27ac signal in each region of the merged bed file was determined for all samples of the respective cell type using multiBigwigSummary (Galaxy Version 3.5.4 + galaxy0) from the deepTools2 suite^77^. Differential H3K27ac in each region was identified using edgeR (Galaxy Version 3.36.0 + galaxy5)^80^ and regions with a p-value ≤ 0.05 were regarded as differential regions. Venn diagrams of differential regions were created using the web application DeepVenn (https://www.deepvenn.com)}. For visualisation of the differential regions, Z-scores were calculated in Excel and heatmaps for the differential groups were plotted using the R package ComplexHeatmap^81^. Alluvial plots were generated using the R package alluvial^82^. Differential regions were analysed using ChIPSeeker (Galaxy Version 1.28.3 + galaxy0)^83^ to annotate the differential regions to genomic elements. Genome coordinates of the differential bed files were converted to the hg38 (GRCh38) assembly using the LiftOver tool of the UCSC Genome Browser^84^. Bed files in the hg38 coordinates were then used to investigate the distance of peaks to the next TSS and the enrichment of the Cut&Tag regions in biological pathways by ChIP-Enrich^85^.

### Statistical analysis

Statistical analysis was performed in GraphPad Prism version 8.0.2 (GraphPad Software, Boston, MA, USA). Detailed statistical methods were described in figure legends. Normality distribution was probed where appropriate (n ≥ 5) using the Kolmogorov–Smirnov test. For comparisons involving two independent variables (e.g., astronaut/control and timepoint), we used two-way ANOVA followed by Sidak’s multiple comparisons test. This approach was chosen for independent groups with matching within variable (time course). For non-normally distributed matched data (i.e. the comparison of H3K27ac signal in different genomic regions), the non-parametric Friedman test was applied, followed by Dunn’s multiple comparisons test.

## Supplementary Information

Below is the link to the electronic supplementary material.

Supplementary Material 1

Supplementary Material 2

## Data Availability

NGS data in this publication are available via the NCBI’s Gene Expression Omnibus via the GEO Series accession number GSE288469.
