# Supplementary material for: Epigenomic profiling of immune cell subtypes reveals H3K27ac-marked stress signatures after long-duration spaceflight
Source: Sci Rep. 2025 Sep 12;15:32445. doi: 10.1038/s41598-025-17930-1 (PMC12432228; doi:10.1038/s41598-025-17930-1)
Supplement: Supplementary file 1 — Supplementary Material 1 [file 41598_2025_17930_MOESM1_ESM.pdf]

Epigenomic profiling of immune cell subtypes identifies stress-associated signatures after long-duration spaceflight

Tabea L Fullstone[1], Lukas Fischer[1], Maria Bohmeier[2], Petra Frings-Meuthen[2], Brian E Crucian[3], Philipp Rathert[1*]

[1] Department of Biochemistry, Institute of Biochemistry and Technical Biochemistry, University of Stuttgart, 70569 Stuttgart, Germany.
[2] German Aerospace Centre (DLR), Institute of Aerospace Medicine, Linder Hoehe, 51147 Cologne, Germany
[3] National Aeronautics and Space Administration, Johnson Space Center, Houston, TX, United States.

[*] To whom correspondence should be addressed. Tel: +49-711-685-64388; Fax: +49-711-685-64392; Email: philipp.rathert@ibtb.uni-stuttgart.de

Supplementary Fig. 1: Gating strategy for the analysis of PBMC subpopulations by antibody staining.

Supplementary Fig. 2: Distribution of PBMC subtypes among total PBMC is not influenced by prolonged orbital spaceflight.

Supplementary Fig. 3: Extraction of PBMC subtypes from total PBMCs using magnetic microbeads.

Supplementary Fig. 4: Astronauts experience early, late and long-term changes in H3K27ac in CD4+ immune cells upon prolonged orbital spaceflight.

Supplementary Fig. 5: Prolonged orbital spaceflight leads to the loss of H3K27ac at promoters and gain at intronic and distal intergenic elements.

Supplementary Fig. 6: Top 10 GO pathways associated with differential peaks of CD8+ immune cells following prolonged orbital spaceflight.

Supplementary Fig. 7: Top 10 GO pathways associated with differential peaks of CD4+ immune cells following prolonged orbital spaceflight.

Supplementary Table 1: Reference ranges for PBMC subtypes in total PBMCs.

Supplementary Table 2: Cell numbers of total PBMCs and PBMC subtypes extracted.

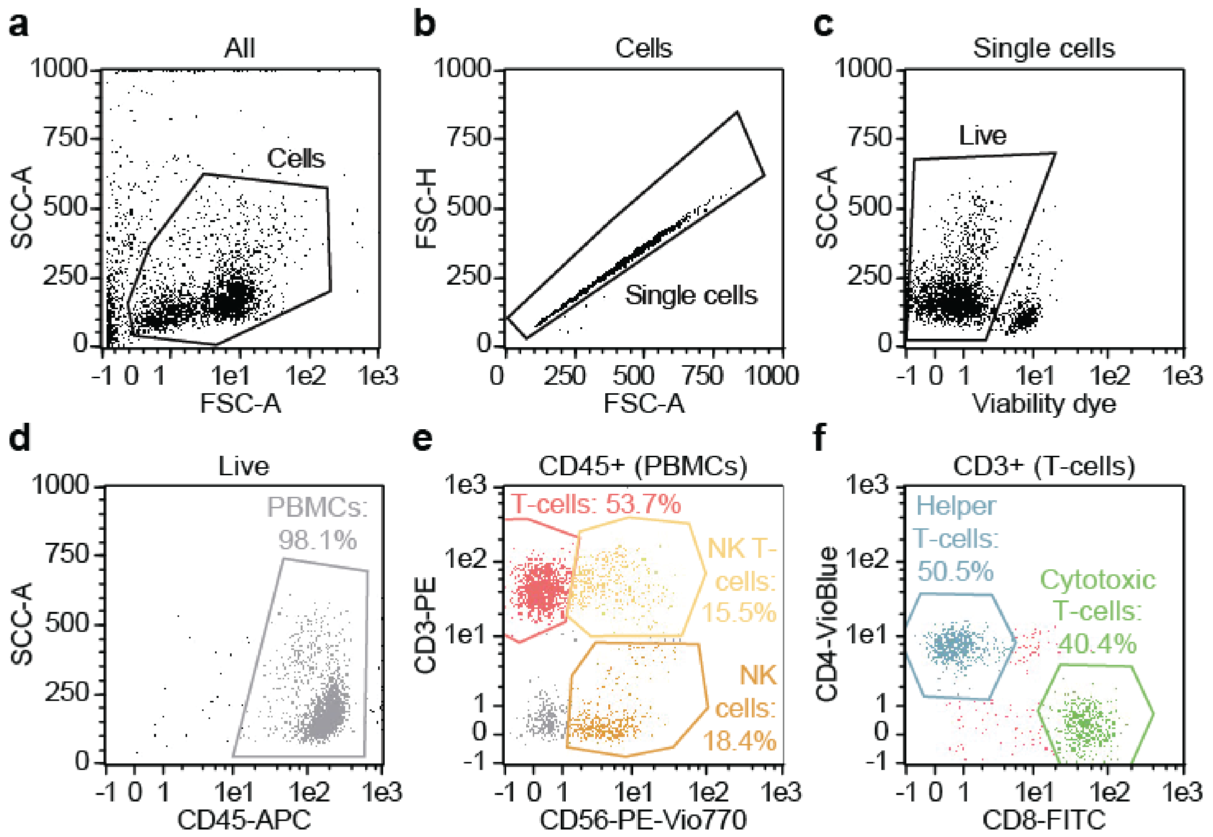

Supplementary Fig. 1: Gating strategy for the analysis of PBMC subpopulations by antibody staining. **(a-f)** Representative antibody staining of PBMCs isolated from whole blood using viability dye and an antibody panel directed against CD45, CD3, CD56, CD4 and CD8. After gating for intact cells **(a)** and removal of doublets **(b)**, living cells were identified by exclusion of a viability dye **(c)**. Living cells were then gated for total PBMCs based on their CD45 expression **(d)** and these further analysed for expression of CD3 and CD56 **(e)** to distinguish T-cells (CD3+ only), natural killer cells (NK cells, CD56+ only) and natural killer T-cells (NK T-cells, CD3+ and CD56+). Finally, T-cells were analysed for their CD4 and CD8 expression levels **(f)** to distinguish between helper T-cells (CD3+ and CD4+) and cytotoxic T-cells (CD3+ and CD8+). Panel shows representative images of n=18 different stainings.

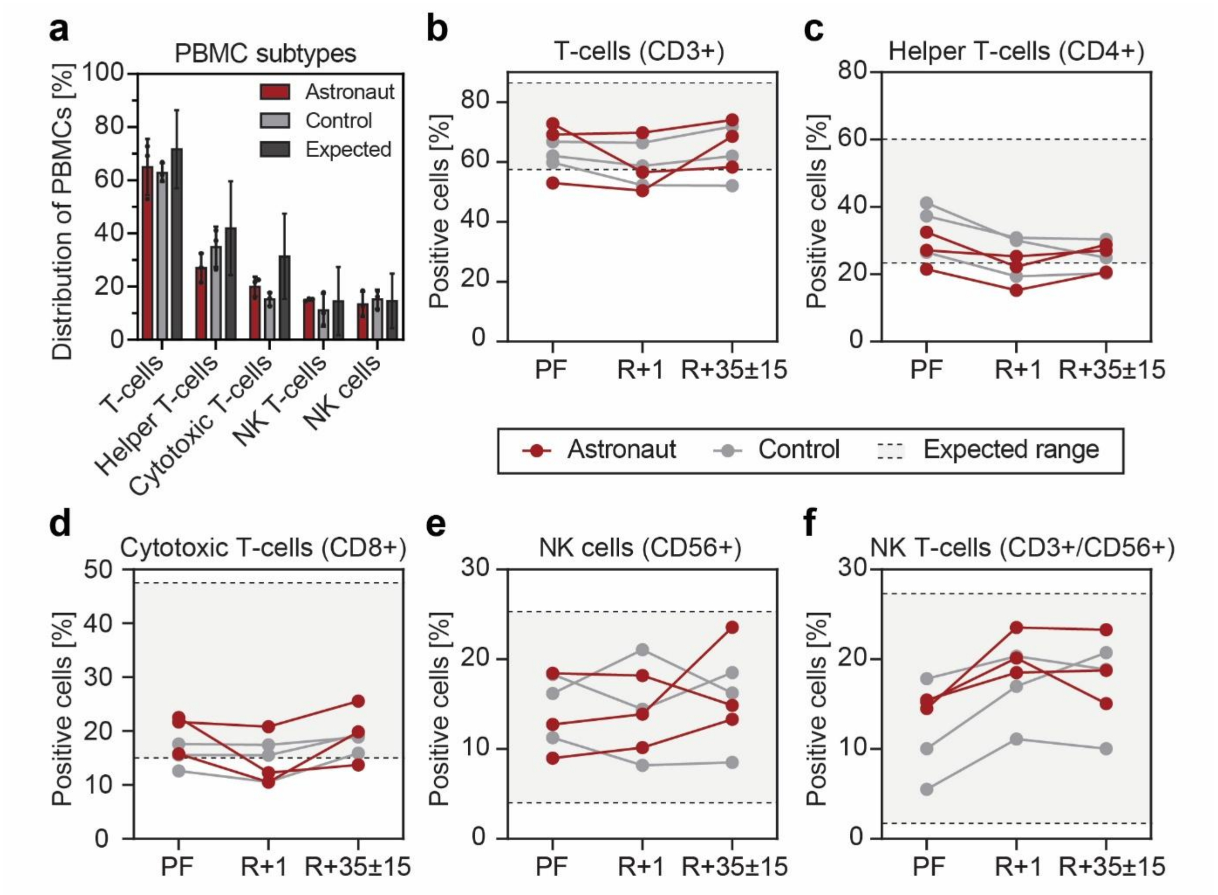

Supplementary Fig. 2: Distribution of PBMC subtypes among total PBMC is not influenced by prolonged orbital spaceflight. (a) Distribution of PBMC subtypes among total PBMCs of astronauts and control subjects pre-flight. Shown is the percentage of each subtype in the total PBMC population. Expected values were taken as an average from five different studies (Supplementary Table 1). Mean±SD, n=3. **(b-f)** Percentage of T-cells **(b)**, helper T-cells **(c)**, cytotoxic T-cells **(d)**, natural killer cells **(e)** and natural killer T-cells **(f)** among total PBMCs for control subjects and astronauts. Total PBMCs for all subjects were extracted and stained pre-flight and post-flight at R+1 and R+35±15. Shown is the percentage of each subtype in the total PBMC population. Expected values for each PBMC subtype (Supplementary Table 1) are indicated between the dashed lines. Statistical significance testing using two-way ANOVA with Sidak's multiple comparisons test yielded no significant results for astronaut samples.

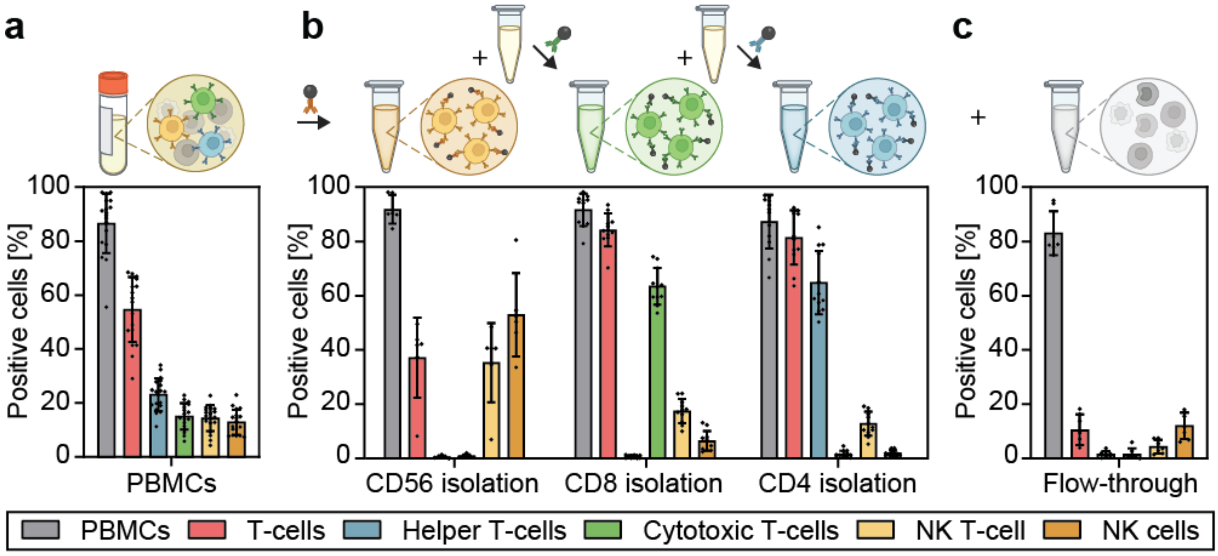

Supplementary Fig. 3: Extraction of PBMC subtypes from total PBMCs using magnetic microbeads. This figure demonstrates the effectiveness of our cell purification method, using magnetic microbeads to isolate specific PBMC subtypes. This methodological control confirms the reliability of our cell separation approach prior to downstream analyses. **(a-c)** Antibody staining of astronaut and control samples before and after isolation of PBMC subtypes. Specific PBMC subtypes were isolated from the total PBMC population using antibody-bound magnetic microbeads in sequential order (CD56 → CD8 → CD4). The original PBMC population **(a)**, isolated PBMC subtypes **(b)** and leftover cells in the flow-through **(c)** were analysed for PBMC distribution by antibody staining. Mean±SD, n=6–18 (PBMCs: n=18, CD56 isolation: n=6, CD8 isolation: n=11, CD4 isolation: n=12, Flow-through n=7).

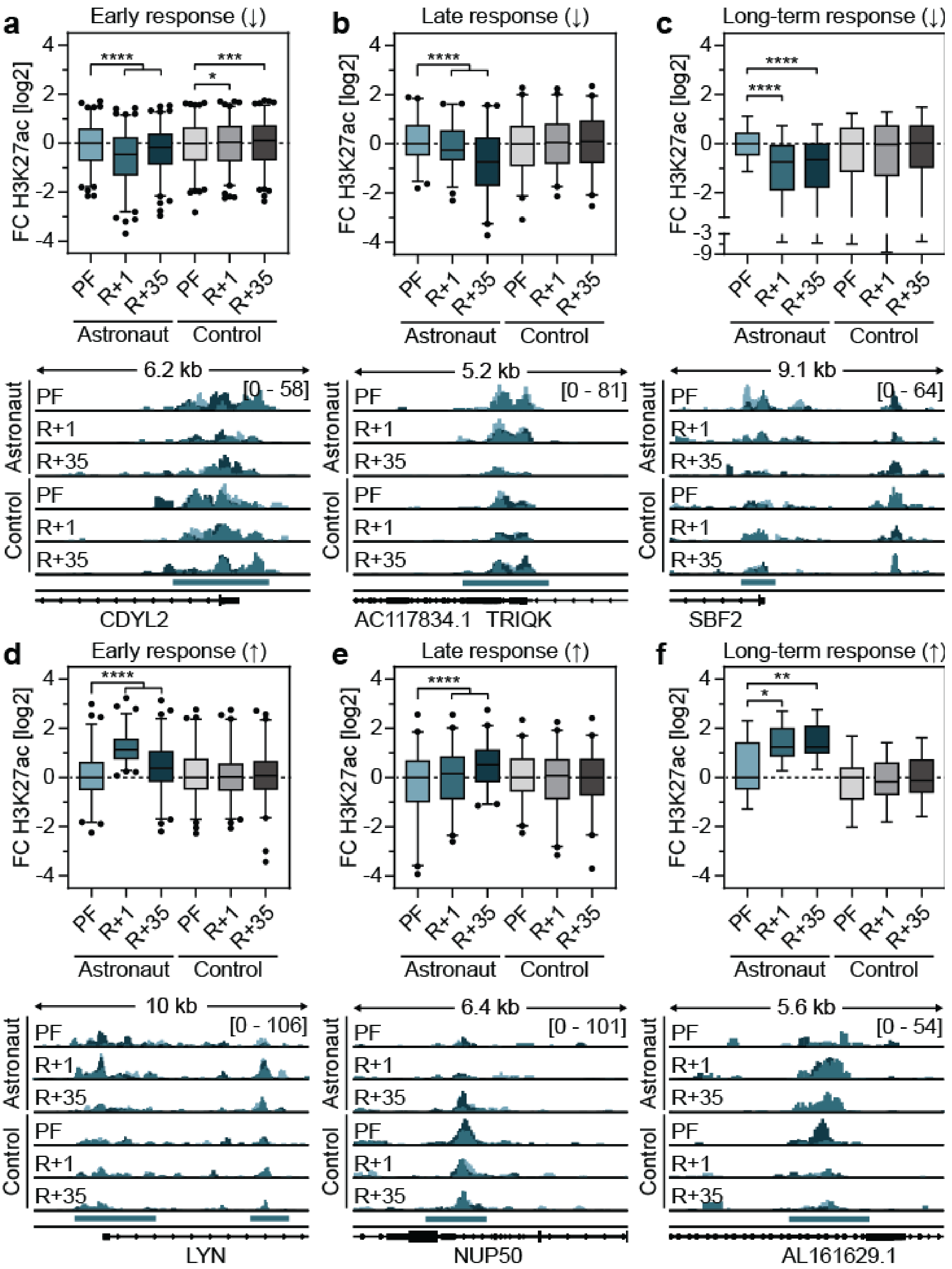

Supplementary Fig. 4: Astronauts experience early, late and long-term changes in H3K27ac in CD4+ immune cells upon prolonged orbital spaceflight. (a-f) Top: Boxplot showing changes in H3K27ac in CD4+ immune cells following prolonged orbital spaceflight. Shown are clusters with loss **(a-c)** or gain **(d-f)** in H3K27ac that show an early response/change in R+1 **(a,d)**, late response/change in R+35 **(b,e)** or long-term response/change in R+1 and R+35 **(c,f).** H3K27ac signal of astronauts and control subjects pre-flight (PF) and post-flight at R+1 and R+35±15 was retrieved from bigwig files. The RPKM

signal of the differential regions was averaged over each replicate and is displayed relative to the median of the PF sample in each group (astronaut or control). Box plot with 2.5 and 97.5 percentiles with individual data points shown only for values falling outside this interval, statistical analysis: Friedman test and Dunn's multiple comparisons test (*: $p \leq 0.05$, **: $p \leq 0.01$, ***: $p \leq 0.001$, ****: $p \leq 0.0001$). Combined bracket signifies same significance for all three comparisons (PF vs R+1 vs R+35). Bottom: Representative Cut&Tag tracks of the boxplot of RPKM normalised H3K27ac intensity in CD8+ immune cells of control subjects and astronauts pre-flight and post-flight at R+1 and R+35±15. All samples were grouped to the same maximum signal indicated in the first line. Each replicate is shown in a different shade of blue and overlaid on the same track. n=3.

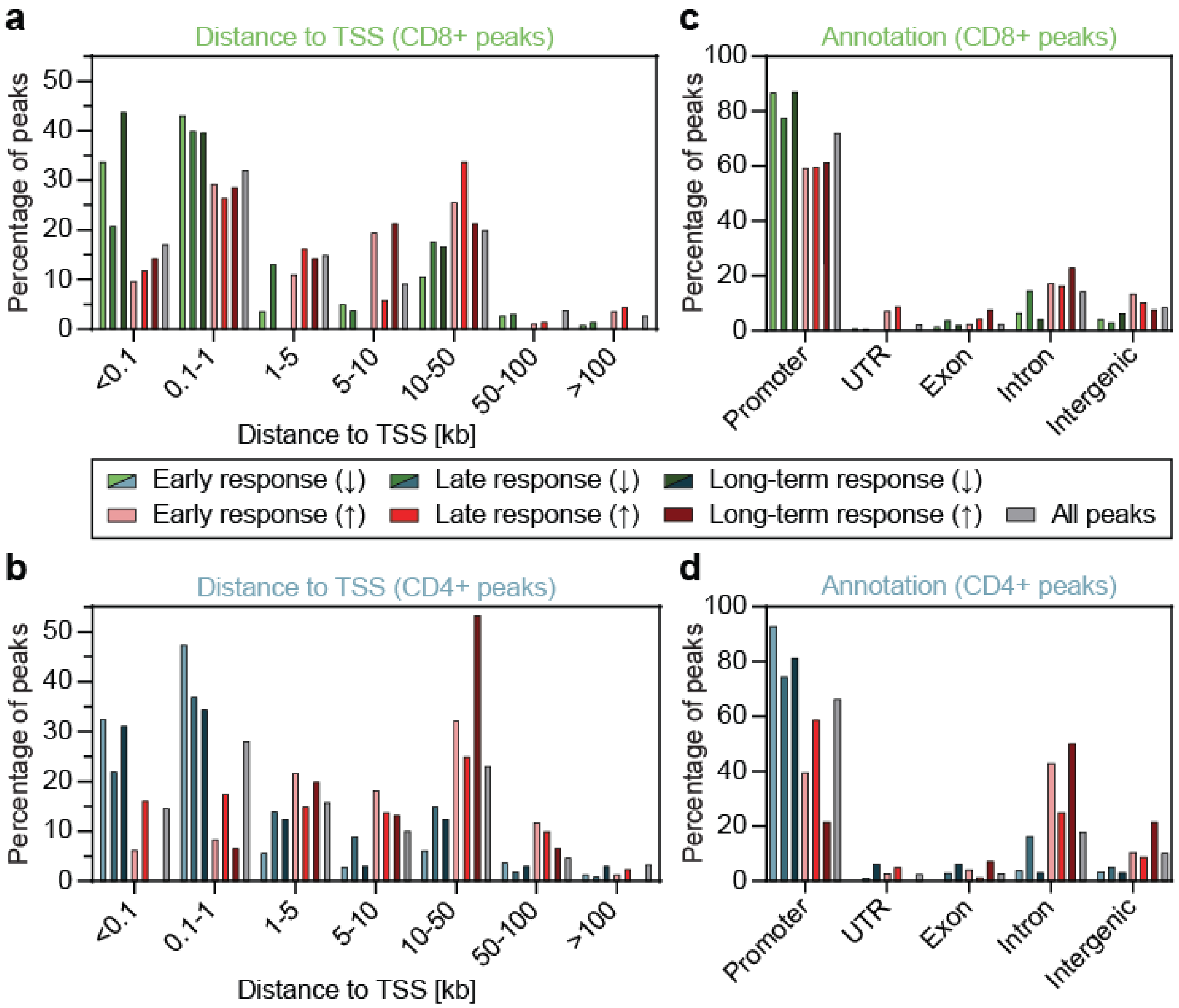

Supplementary Fig. 5: Prolonged orbital spaceflight leads to the loss of H3K27ac at promoters and gain at intronic and distal intergenic elements. (a and **b)** Distance of the differential H3K27ac regions from CD8+ (a) or CD4+ **(b)** immune cells to the nearest transcription start site (TSS) identified by ChIP-Enrich. **(c** and **d)** Annotation of differential H3K27ac regions in CD8+ **(c)** or CD4+ **(d)** immune cells to different genomic elements identified by ChIPSeeker. Shown is the percentage of peaks annotated to promoters (≤1–3 kb), untranslated regions (5' and 3' UTR), exons, introns and distal intergenic elements.

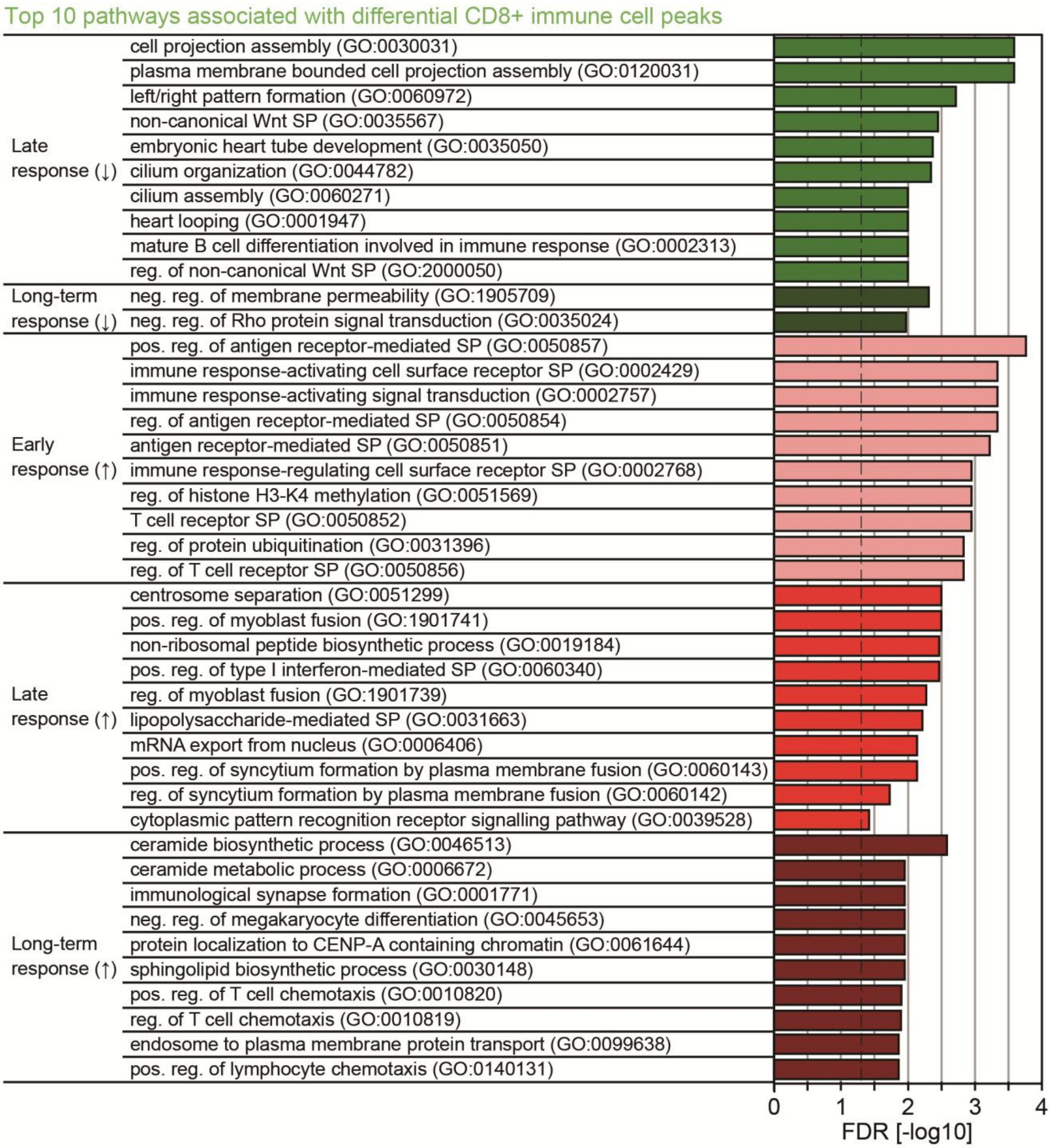

Supplementary Fig. 6: Top 10 GO pathways associated with differential peaks of CD8+ immune cells following prolonged orbital spaceflight. Gene set enrichment analysis for peaks with differential H3K27ac in CD8+ immune cells following prolonged orbital spaceflight. Analysis was performed for GO biological process pathways using ChIP-Enrich. The 10 most significantly enriched pathways are shown with a significance threshold of FDR ≤0.05 (indicated by the dotted line). Panels show pathways associated with H3K27ac peaks that lose (green) or gain (red) H3K27ac either early, late or long-term upon prolonged orbital spaceflight. Abbreviations: SP: signalling pathway, neg: negative, pos: positive, reg: regulation.

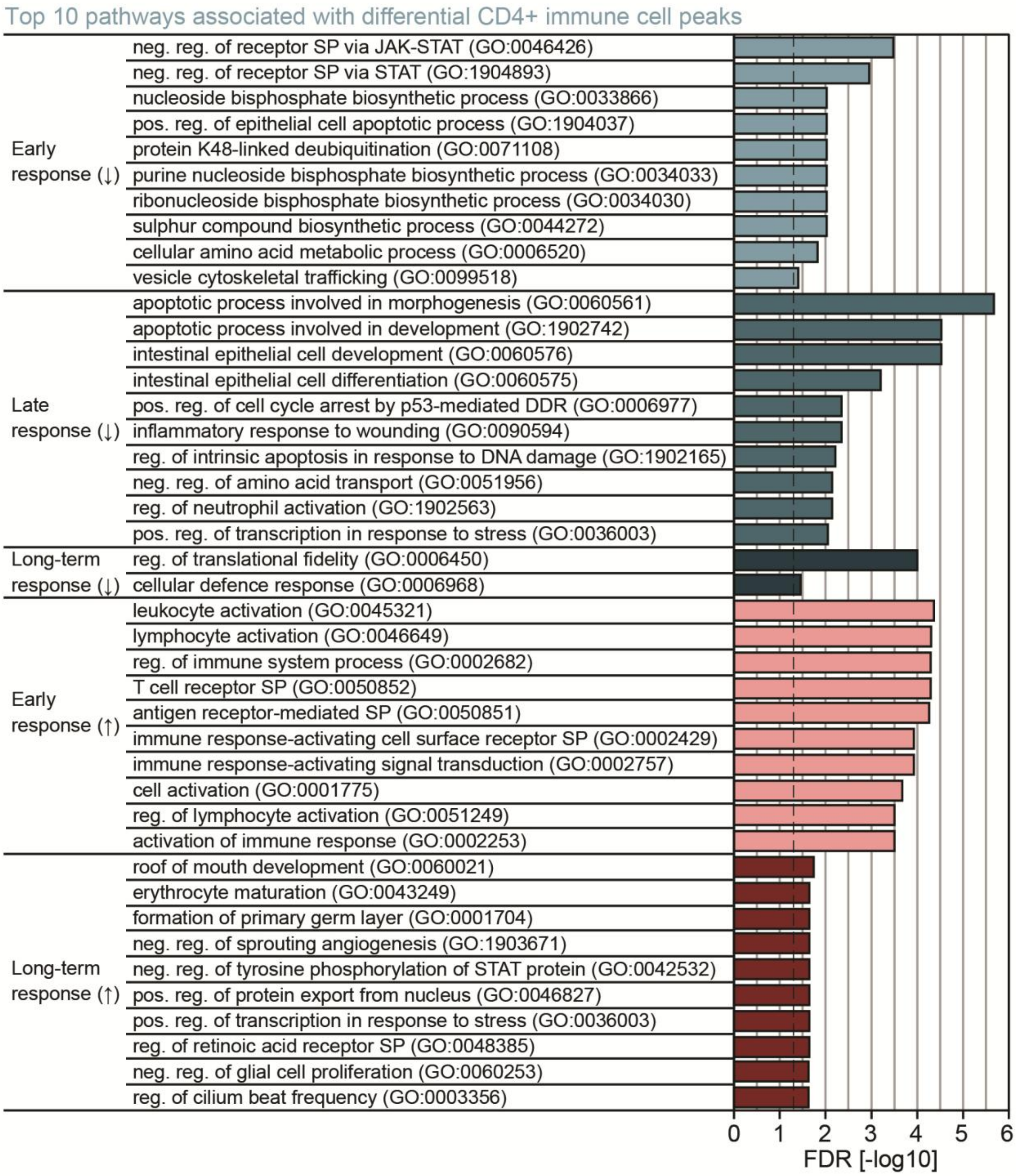

Supplementary Fig. 7: Top 10 GO pathways associated with differential peaks of CD4+ immune cells following prolonged orbital spaceflight. Gene-set-enrichment analysis for peaks with differential H3K27ac in CD4+ immune cells follow6ing prolonged orbital spaceflight. Analysis was performed for GO biological process pathways using ChIP-Enrich. The 10 most significantly enriched pathways are shown with a significance threshold of FDR ≤0.05 (indicated by the dotted line). Panels show pathways associated with H3K27ac peaks that lose (blue) or gain (red) H3K27ac either early, late or long-term upon prolonged orbital spaceflight. Abbreviations: SP: signalling pathway, neg: negative, pos: positive, reg: regulation.

Supplementary Table 1: Reference ranges for PBMC subtypes in total PBMCs.

Reference ranges for PBMC subtypes in healthy adults were collected from different literature sources. Studies with different origins, including Hungary (HU), USA (US), Belgium (BE), Sweden (SE), the United Kingdom of Great Britain (GB), Spain (ES) and Brazil (BR) were chosen to avoid country-specific bias. The mean range for each subtype (avg.) was determined to better represent subjects with different nationalities.

Study origin	T-cells (CD3+)	Helper T-cells (CD3+ CD4+)	Cytotoxic T-cells (CD3+ CD8+)	NK T-cells (CD3+ CD56+)	NK cells (CD3- CD56+)	Ref.
**HU**	66.1 – 75.5%	33.3 – 44.6%	21.5 – 36.4%	3.5 – 7.0%	5.6 – 15%	{Koreck et al. 2002}
**US**	65.0 – 88.0%	26.0 – 62.0%	14.0 – 44.0%	–	2.0 – 27.0%	{Valiathan et al. 2014}
**US, BE, SE, GB**	61.0 – 85.0%	28.0 – 58.0%	19.0 – 48.0%	–	6.0 – 29.0%	{Reichert et al. 1991}
**ES**	41.6 – 99.8%	10.2 – 79.2%	9.5 – 68.7%	0.7 – 53.8%	–	{Andreu-Ballester et al. 2012}
**BR**	51.3 – 83.5%	24.4 – 54.2%	12.8 – 40.2%	0.9 – 21.4%	3.7 – 28.5%	{Rudolf-Oliveira et al. 2015}
**Avg.**	57.0 – 86.4%	24.4 – 59.6%	15.4 – 47.5%	1.7 – 27.4%	4.3 – 24.9%	

Supplementary Table 2: Cell numbers of total PBMCs and PBMC subtypes extracted. Total PBMCs and PBMC subtypes were isolated from whole blood or three astronauts and three control subjects at three different time points (PF, R+1 and R+35±15). Total PBMCs were isolated by density gradient centrifugation, PBMC subtypes were isolated from total PBMCs using antibody-bound magnetic microbeads in sequential order (CD56 → CD8 → CD4). Given are the medians of total cell numbers $\times 10^5$ with the range (Min – Max) across all three time points.

	**Total PBMCs**	**CD56 isolation**	**CD8 isolation**	**CD4 isolation**
**Astronauts**	22.05 (13.46 – 50.46)	0.73 (0.57 – 4.65)	1.08 (0.59 – 7.72)	0.9 (0.35 – 8.22)
**Controls**	32.77 (12.19 – 82.24)	1.46 (0.45 – 3.06)	2.35 (0.58 – 6.54)	4.29 (1.41 – 9.53)
**Combined**	22.47 (12.19 – 82.24)	1.01 (0.45 – 4.65)	1.59 (0.58 – 7.72)	2.1 (0.35 – 9.53)
